# How and when does responsible leadership affect employees’ pro-environmental behavior?

**DOI:** 10.3389/fpsyg.2022.1079720

**Published:** 2022-12-22

**Authors:** Yuanyuan Zhou, Yumei Ning, Hui He, Dan Li

**Affiliations:** ^1^School of Business, Xinyang Normal University, Xinyang, China; ^2^School of Business Administration, Zhongnan University of Economics and Law, Wuhan, China; ^3^Business School, Yulin Normal University, Yulin, China

**Keywords:** responsible leadership, perceived organizational support toward the environment, green self-efficacy, promotion focus, prevention focus, employee pro-environmental behavior

## Abstract

By integrating the resource accumulation perspective and the Job Demands-Resources (JD-R) model, this research explores the impact mechanism of responsible leadership on employees’ pro-environmental behaviors (EPB). We conducted a survey to collect data from 319 employees in Chinese companies in three waves. Our research found that responsible leadership had significantly positive effect on EPB; employees’ perceived organizational support toward the environment (POS-E) and green self-efficacy played a serial multiple mediating role; meanwhile, employees’ chronic regulatory focus moderated the relationship between responsible leadership and EPB, with promotion focus strengthening the relationship and prevention focus weakening the relationship. Our findings support the applicability of JD-R model in the field of EPB, enrich the theoretical research on responsible leadership and EPB, and also provide a practical basis for organizations to effectively stimulate EPB and promote sustainable development.

## 1. Introduction

The deteriorating ecological environment, the constraints of external environmental regulations, the awakening social awareness of environmental protection, and the cost disadvantages amid fierce competition all require companies to operate sustainably in a way that protects the environment and conserves resources. The sustainable development of the organization must rely on the recognition and participation of employees, and the way of employee participation will determine the progress of an organization’s achievement of its sustainable development goals. Studies have shown that employee is a key resource for organizational environmental practices (Buysse and Verbeke, 2003) and employee behavior is an important component of successful environmental activities of the organization (Boiral, 2009; Wu and Pagell, 2011). Therefore, involving employees in environmental activities directly has become a challenge for organizations (Sarkis et al., 2010). EPB refers to the environmental practice adopted by employees in the organization (Kim et al., 2017), including waste recycling and reuse, adopting sustainable working methods, developing green processes and products, and learning green knowledge (Jenny et al., 2017). This behavior can help companies reduce costs, establish a positive image, and achieve sustainable development initiatives (Faraz et al., 2021). EPB is not a mandatory behavior of the organization, but a proactive behavior of the employee (Cantor et al., 2015). Therefore, external environmental factors are needed to stimulate such behavior.

Leader act as an important source of information for employee and can have a significant impact on employee behavior (Leroy et al., 2018). Leadership has been considered a significant predictor variable of EPB. Much of the existing research has focused on the impact of transformational leadership (Graves et al., 2013; Cop et al., 2020), spiritual leadership (Afsar et al., 2016) and servant leadership (Faraz et al., 2021) on EPB. As an emerging leadership, in addition to role modeling and caring for employees characterized by leadership above, responsible leadership acts more as a coordinator of multiple stakeholder interests. It emphasizes positive social communication and sustainable value creation (Maak and Pless, 2006; Haque et al., 2019b), and is more aligned with the environmental goals of the organization. It brings more resources related to environmental practices and provides another insight to promote EPB (Miska and Mendehall, 2018). In addition, Chinese enterprises have begun to explore the practice of responsible leadership. For example, COFCO Corporation, as the leader of the agricultural and food industry in China, is committed to cultivating responsible managers. Through delicacy management, COFCO actively promotes paperless office, double-sided paper utilization, green workstations, and calls on employees to fulfill their environmental responsibilities. Under the guidance of responsible leader, China’s top-selling dairy firm Yili Group strengthens green management, actively cultivates carbon-neutral talents, and guides employees to integrate the concept of environmental protection into their daily work. Then, will responsible leadership really affect EPB? If so, how does responsible leadership affect EPB? Whether based on theory or practice, it is necessary for us to explore these issues in depth. However, the current research on responsible leadership and EPB is still at the initial stage, previous studies are conducted from the perspective of social identity theory and social exchange theory (Han et al., 2019; Afsar et al., 2020; Xiao et al., 2021; Zhang J. W. et al., 2021), neglect the motivational process of enhancing employees’ resources, and lack in-depth exploration on employees’ potential psychological mechanism. Moreover, previous studies seem to ignore the role of individual differences in the impact of responsible leadership. To fill this gap and respond to the call to explore the bridge that links responsible leadership and employee behavior (Doh and Quigley, 2014), we will explore the role of job resource, individual psychological resource, and individual characteristics resource in the impact of responsible leadership on EPB from the perspective of resources according to JD-R theory.

A growing body of research has emphasized the importance of resource in examining leaders’ influence on employees’ behaviors (Breevaart et al., 2013). The JD-R model indicates that excessive job demands will cause job burnout, which is called “fatigue process”; abundant job resources will bring higher work engagement, which is called “motivational process” (Hakanen et al., 2008). Xanthopoulou et al. (2007) further introduced personal resources (e.g., self-efficacy) into the JD-R model, including psychological resources, competence resources, etc. This study will combine the resource accumulation perspective and the motivational processes of the JD-R model to explore the mediating mechanism by which responsible leadership influences EPB. As resources accumulate, those generated by responsible leadership can help employees to increase other resources, such as job and personal resources (Demerouti et al., 2001; Schaufeli and Bakker, 2004; Xanthopoulou et al., 2007). According to the motivational processes of the JD-R model, job resources and personal resources are the key factors to influence employees’ work behaviors. In other words, leaders shape the work environment of their employees (Smircich and Morgan, 1982), influence their job and personal resources, and ultimately their proactive behaviors (Oprea et al., 2020). According to previous studies (Lamm et al., 2015; Faraz et al., 2021; Paillé and Valeau, 2021), POS-E and green self-efficacy are important job resource and personal resource that affect EPB, respectively. Therefore, they are selected as the psychological mechanisms of responsible leadership to affect EPB in our study. Based on this logic, help and attention offered to employees by responsible leaders in environmental practices may increase employees’ POS-E, which in turn leads to EPB; responsible leaders’ communication and guidance to employees may stimulate employees’ green self-efficacy, which in turn triggers EPB. Therefore, POS-E and green self-efficacy may mediate the relationship between responsible leadership and EPB. In addition, according to the resource accumulation perspective and the JD-R model, personal resources may play a mediating role between job resources and employee outcomes (Xanthopoulou et al., 2007). Therefore, our research will further explore the serial mediating effects of POS-E and green self-efficacy between responsible leadership and EPB.

Not all employees perceive leadership in the same way, and the impact of leadership on employees’ behaviors can be influenced by individual characteristics of employees. Individual characteristics can also be considered as personal resources (Halbesleben et al., 2009). The JD-R model demonstrates that personal resources may play a moderating role between environmental factors and employee outcomes and influence the way employees understand and respond to the environment (Xanthopoulou et al., 2007). Responsible leadership, as an environmental factor, will help employees to obtain more resources, and may also trigger the depletion of employees’ own resources because of the expectations and demands placed on them (Zhu et al., 2021). Individual characteristics that tend to focus on resource acquisition and positive outcomes enhance the positive effect of responsible leadership on pro-environmental behaviors, while those that tend to focus on resource loss and negative outcomes weaken the positive effect. Therefore, we select the promotion and prevention focus of employees (Higgins, 1997) to explore their moderating role between responsible leadership and EPB.

**FIGURE 1:**
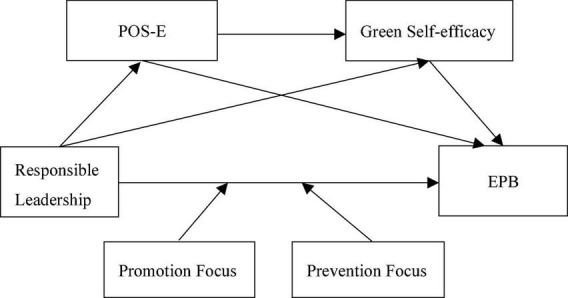
Theoretical model.

Taken together, our study makes several important contributions. First, based on JD-R theory, POS-E and green self-efficacy are cast to explain the relationship between responsible leadership and EPB, which enriches the antecedents of EPB, provides a novel perspective on how responsible leadership affects EPB, and deeply explored the psychological motivation mechanism based on Chinese context. Second, our study introduces the regulatory focus as an important boundary condition for the effects of responsible leadership, which provides new insights into the contingency relationship between responsible leadership and EPB. Third, our study supports the JD-R theory, which broadens the application scope of this theory. Figure 1 depicts the overall theoretical model.

## 2. Theory and hypotheses

### 2.1 Responsible leadership and EPB

As the business environment changes, organizations face more social, ethical and environmental challenges, which require leaders to take on more responsibility for building the trust of society in themselves and the organizations. This has led to the birth of responsible leadership, which is now a necessity for the survival and success of the organization. Previous research on leadership has focused on the binary leader-employee interaction, which is no longer appropriate for the current global business environment. In contrast, responsible leadership focuses not only on stakeholders within the organization (e.g., employees), but also on stakeholders outside the organization (e.g., government, suppliers, consumers, etc.), considers the impact of the actions to be taken on all stakeholders, and exerts the influence by engaging affected stakeholders in consultation (Voegtlin, 2011). According to Pless and Maak (2011), responsible leadership focuses on social and environmental goals and sustainable value creation, following the “triple bottom line”, aligning corporate profitability, social responsibility, and environmental responsibility. Responsible leadership seeks harmony between people and nature, which is consistent with the philosophy of EPB. At the individual level, responsible leadership leads to higher employee job satisfaction (Voegtlin, 2011); it also enhances employee pride (Doh et al., 2011), boosts employees’ emotional commitment (Haque et al., 2019a), creates an ethical climate, and thus enhances corporate image (Yasin, 2021), ultimately reduces employees’ willingness to leave and helps companies retain their talent.

Employees pro-environmental behaviors refers to the positive and proactive environmentally friendly behavior exhibited by employees in their work practices (Graves et al., 2013), which can both reduce organizational costs and directly benefit the organization, as well as contribute to the organization’s efforts to protect natural resources and the environment, fulfill corporate social responsibility, and achieve sustainability. Ying et al. (2020) noted that in many companies, EPB does not appear in job descriptions and are not monitored or rewarded by the organization. They are the pro-social and extra-role behaviors of most employees, and are non-coercive and voluntary in nature.

Leadership is an important factor influencing employee behavior, and leaders’ behavior can influence followers’ motivation and pro-social behavior (Ilies et al., 2007; Derue et al., 2011). Tepper et al. (2018) indicate that the process of leader-employee interaction can be seen as a process in which leaders provide resources to employees. From the perspective of resource accumulation, responsible leaders can provide resources to employees, including personal characteristics resources, conditional resources, relationship resources and so on (Hobfoll, 2002), and employees will strive to maintain these resources and take positive actions to achieve future resource appreciation, and EPB is one of the behaviors that employee adopts to realize resource appreciation, which helps to enhance employee well-being and long-term development in the organization (Zhang B. et al., 2021). Firstly, in terms of personal characteristics resources, responsible leaders consider the affected stakeholders before making business decisions, accommodate different views of various stakeholders inside and outside the organization to seek balanced decisions, and solve problems through consulting; they take more responsibility for work, are highly competent with good moral standards. As a result, responsible leaders are respected and trusted by employees and are seen as moral role models worthy of emulation (Voegtlin et al., 2019), which in turn prompt pro-environmental behaviors. Secondly, in terms of conditional resources, responsible leaders attach importance to the impact of corporate decisions on society and the natural environment, make the fulfillment of corporate social responsibility a goal of organizational development (Pless, 2007), and convey environmental values to employees through their own actions. These behaviors resonate deeply within employees. Responsible leaders also focus on the long-term development of employees, create the conditions for employees to achieve their goals and personal accomplishments, and finally lead to employees’ positive behaviors. Thirdly, in terms of relational resources, responsible leaders care about the needs of their subordinates, involve employees in the decision-making process (Doh and Quigley, 2014), care for and trust their employees (Brown et al., 2005), which lead to recognition and gratitude among employees. At the same time, employees make efforts to express values similar to those of their leaders and engage in pro-environmental behaviors in order to build and strengthen relationships with their leaders (Kim et al., 2017). Therefore, we propose:

Hypothesis 1 (H1): Responsible leadership has a positive impact on EPB.

### 2.2 The mediating role of green self-efficacy

Self-efficacy is the degree of confidence people have in their ability to perform a task or action using the skills they possess (Bandura, 1977). According to the JD-R model, self-efficacy is a typical personal psychological resource (Xanthopoulou et al., 2007). Self-efficacy associated with a specific domain can be a more effective predictor of behavior in that domain (Choi, 2004). In the current context of ecological environmental protection, Chen et al. (2015) and Kim et al. (2016) introduced the concept of self-efficacy to the field of environmental protection and proposed the concept of green self-efficacy. Green self-efficacy refers to an individual’s assessment and judgment of their ability to achieve environmental goals. Prior studies have indicated the important role of employee self-efficacy in promoting positive behaviors (e.g., pro-environmental behaviors) (Lee et al., 2014; Abraham et al., 2015; Huang, 2016; Kim et al., 2016).

Leadership, as a work-related environmental factor, can motivate and enhance employees’ personal resources (Wegge et al., 2014). It has been shown that employees’ self-efficacy is related to the behaviors of their leaders (Ren and Chadee, 2017). By communicating with employees, responsible leaders keep abreast of their needs and difficulties, help stimulate their potential, convince them to overcome current challenges, and enhance their self-efficacy. Responsible leaders encourage information sharing, provide a platform for stakeholder consultation and communication, and facilitate knowledge sharing among environmental stakeholders (Voegtlin et al., 2012). Responsible leaders themselves act as intermediaries of information and knowledge, they have the ability to acquire and disseminate knowledge related to organizational sustainability and environmental protection (Doh and Quigley, 2014). This knowledge-sharing process allows employees to acquire the necessary environmental knowledge, skills and abilities which help enhance their green self-efficacy. Responsible leaders also attach importance to coaching and training employees in environmental protection and sustainable development, reward “pioneers of environmental protection,” and help them develop a broader understanding of corporate responsibility in society (Maak and Pless, 2006). Therefore, responsible leadership can help enhance employees’ green self-efficacy.

According to the JD-R model, employees with high self-efficacy usually have abundant personal psychological resources (e.g., confidence, courage) and believe they are capable of completing challenging tasks. Individuals with higher levels of self-efficacy are more likely to have higher performance and satisfaction, more commitment and work engagement (Bakker et al., 2008; Luthans et al., 2008), and more likely to engage in pro-social behavior (Grant and Gino, 2010). Research has shown that high levels of self-efficacy can promote proactive behaviors (Huang, 2017), help employees face difficulties and accomplish complex behaviors, e.g., EPB (Lo et al., 2012; Paillé et al., 2019b). Green self-efficacy activates employees’ environmental beliefs and attitudes, helps them feel more competent and confident in performing tasks related to environmental protection. Employees with high green self-efficacy will take the initiative to acquire new environmental knowledge, be courageous in solving problems in environmental practices, and actively engage in pro-environmental behaviors. On the contrary, when employees’ green self-efficacy is low, employees lack the confidence and ability to participate in green practices, which may reduce their willingness toward pro-environmental behaviors. Therefore, we propose:

Hypothesis 2 (H2): Green self-efficacy mediates the relationship between responsible leadership and EPB.

### 2.3 The mediating role of POS-E

According to Lamm et al. (2015), POS-E is a perception by employees of how the organization evaluates their contribution to sustainability, including the belief that the organization provides opportunities for environmental practices and pays attention to their environmental contributions (Paillé and Valeau, 2021). POS-E represents employees’ perceptions of the extent to which the organization values sustainability efforts. According to the JD-R model, POS-E is a job resource for employees.

Leaders in organizations are often seen by employees as representatives of the organization, and the way leaders treat employees is seen by employees as the way the organization treats them. Responsible leaders as supportive leaders (Maak and Pless, 2006), fully consider employees’ personal needs before making decisions, strive to build a fair work climate (Antunes and Franco, 2016), and make employees aware that they are valued (Voegtlin, 2011). The ethical orientation of responsible leaders drives them to care about their employees (Brown et al., 2005), to pay attention to the difficulties that employees encounter in environmental practices, to guide environmental practices, to help employees understand and adhere to environmental policies, and to give employees emotional and instrumental support. Responsible leaders can assess the impact of business decisions on the natural environment and ensure that production processes are as environmentally friendly as possible (Maak and Pless, 2006). They are committed to environmental practices, actively involved in the ecological transformation of the workplace, communicate and share information with environmental stakeholders, pay attention to the needs and interests of environmental stakeholders (Pless and Maak, 2011), therefore employees may feel supported by the organization. That is, the responsible leaders care, help and guide their subordinates so that employees perceive the organizational support in environmental protection and their job resources are increased.

The accumulation of job resources will help to enhance employee initiative and spontaneity at work (Zacher et al., 2019). On the one hand, POS-E can satisfy employees’ sense of belonging in the organization and their sense of accomplishment in engaging in environmental activities. Employees’ emotional needs are fulfilled, and they are motivated to voluntarily adopt behaviors that benefit the organization. Prior research has indicated that when employees perceive organizational support for a particular initiative or practice (e.g., environmental protection), they will show more engagement and satisfaction, thus demonstrate higher levels of voluntary commitment to the goal (Grant et al., 2008; Bingham et al., 2013), prompting employees to engage in pro-environmental behaviors in their daily work (Paillé and Boiral, 2013). On the other hand, when the organization provides employees with environmental support, employees will engage in pro-environmental behaviors in return for the support of organization. Employees will have less willingness to engage in EPB without being aware of whether they will be appreciated or supported by the organization (Lamm et al., 2015). As a result, employees use POS-E as a guide to make decisions about pro-environmental behaviors. Therefore, we propose:

Hypothesis 3 (H3): POS-E mediates the relationship between responsible leadership and EPB.

### 2.4 The serial mediating role of POS-E and green self-efficacy

According to the JD-R model, job resources (e.g., POS-E) may activate personal resources (e.g., green self-efficacy), leading to positive outcomes (Xanthopoulou et al., 2007). Perceived organizational support is an important factor that impacts self-efficacy (Luthans et al., 2008), and POS-E creates positive conditions for stimulating employees’ psychological potential in environmental practices. On the one hand, instrumental environmental support can help improve employees’ green self-efficacy. The organization provides employees with opportunities and resources to participate in environmental practices, and affords coaching and training on the knowledge and skills required to use in environmental practices, which can further enhance employees’ confidence in implementing environmental tasks. On the other hand, emotional environmental support can help improve employees’ green self-efficacy. When employees feel that the organization cares, encourages and praises them for their environmental practices, they will be more proactive in their work in order to repay the organization’s affirmation and care (Korsgaard et al., 2010), and this positive emotional state is conducive to improving employees’ green self-efficacy. In addition, POS-E brings employees closer to their leaders and colleagues, making it easier for them to observe and learn from the experiences of others who are engaged in environmental practices, thereby strengthening their beliefs in implementing pro-environmental behaviors. Therefore, we predict that POS-E may enhance employees’ green self-efficacy.

Combining hypotheses 2 and 3, we argue that responsible leaders support and help employees in environmental practices and enhance employees’ POS-E, employees’ green self-efficacy would be relatively higher in an environment with higher POS-E, high employees’ green self-efficacy will further stimulate EPB. Therefore, we propose:

Hypothesis 4 (H4): POS-E and green self-efficacy play a serial mediating role in the relationship between responsible leadership and EPB.

### 2.5 The moderating role of chronic regulatory focus

Chronic regulatory focus is a tendency of individuals to self-regulate in the face of external circumstances (Higgins, 1997, 1998). The regulatory focus theory indicates that individuals have two different regulatory focus in achieving their goals, promotion focus and prevention focus. Lanaj et al. (2012) argued that individuals may have two types of regulatory focus at different degrees at the same time, and the dominant regulatory focus will exert greater impact. Promotion-focused individuals are more focused on growth and development needs and will adopt positive, proactive strategies to achieve positive outcomes. Prevention-focused individuals are more concerned with safety needs and will adopt cautious, avoidant strategies to minimize negative outcomes (Higgins et al., 2001). Thus, the regulatory focus reflects individual characteristics differences at the cognitive level. According to the JD-R model, individual characteristics differences are personal resources that affect individuals’ perceptions of the external environment and information (Ten Brummelhuis et al., 2011; Van den Broeck et al., 2011; Demerouti et al., 2012). Individuals with different regulatory focus may also differ in their interpretations of environmental factors (e.g., leadership type) and behavioral strategies. Prior research has shown that promotion focus enhances the positive effect of ethical leadership on employee work engagement (Cheng et al., 2014), strengthens the effect of leader inclusiveness on employee taking charge behavior (Li et al., 2019), and enhances the positive effect of leader consideration on employee task performance (Choi et al., 2020). Prevention focus weakens the positive impact of the leaders’ initiating structure on employee task performance (Choi et al., 2020). Therefore, chronic regulatory focus, as an individual characteristic variable, may influence the extent to which responsible leadership becomes effective. However, the moderating role of individual chronic regulatory focus has not been given enough attention in the influence of responsible leadership on employee behavior.

Promotion-focused employees tend to perceive their environment from a positive perspective of growth and development. On the one hand, prior research reveals that promotion-focused employees have a more positive perception of acquisition to potential resources (Koopman et al., 2016). Responsible leadership considers the long-term goals of the organization, focuses on the needs and interests of employees, supports their personal growth and career development, and encourages ethical behaviors. Promotion-focused employees are likely to view these behaviors as positive support and believe that responsible leadership can meet their needs, help them achieve goals. Thus, employees’ positive state may be enhanced (Higgins, 2005), the effect of responsible leadership is amplified. On the other hand, the goals of promotion-focused employees match the values proposed by responsible leadership, and the regulatory focus of both fits. According to the interpersonal regulatory fit theory, the interpersonal regulatory fit contributes to enhancing motivation and affects the individual’s work behavior (Righetti et al., 2011). Johnson et al. (2017) found that the leader-follower regulatory focus fit was positively related to employees’ citizenship behaviors. Therefore, promotion-focused employees are more likely to identify with responsible leaders, and generate positive work behaviors (e.g., pro-environmental behaviors).

Prevention-focused employees tend to perceive their environment from a negative perspective of loss avoidance. Prevention-focused employees are more cautious and focus on meeting basic job requirements, fulfilling job duties and responsibilities and avoiding mistakes and risks (Higgins, 1997; Lanaj et al., 2012). Thus, prevention-focused employees are less proactive in nature (Kaplan and Berman, 2010), whereas pro-environmental behavior is proactive and voluntary behavior. Responsible leadership focuses not only on economic responsibility, but also on social and environmental responsibility (Miska et al., 2014). Responsible leadership takes into account the interests of all stakeholders, of which employee is one important part. Responsible leadership may require employees to understand the needs of external stakeholders, communicate well with them, and help resolve conflicts among stakeholders. These requirements may go beyond employees’ daily job responsibilities and increase their stress (Zhu et al., 2021). In addition, prevention-focused employees may worry about “more work, more mistakes” and feel insecure (Adams and Tyler, 2021). Therefore, prevention-focused employees are likely to form negative perceptions of responsible leadership, believing that such leadership will result in the loss of their own resources, and adopt preventive avoidance strategies. This would make the role model of responsible leadership less effective and weaken the impact of such leadership on EPB. Based on this, we propose the following hypotheses:

Hypothesis 5 (H5): Promotion focus strengthens the relationship between responsible leadership and EPB. The relationship is more positive when promotion focus is high rather than low.

Hypothesis 6 (H6): Prevention focus weakens the relationship between responsible leadership and EPB. The relationship is more positive when prevention focus is low rather than high.

## 3. Materials and methods

### 3.1 Procedure and participants

Our primary sample was comprised of full-time employees from the manufacturing, construction, and logistic industries in China, which were closely related to environmental protection. The researchers utilized their personal social networks to contact participants via email and instant messenger and send them links to online questionnaires. We also encouraged these recipients to forward the survey links to colleagues to help us recruit additional participants, as in snowball sampling. In order to ensure the quality of samples, the participants must meet the following conditions: participants were over 18 years of age, working full-time within an organization, could provide effective enterprise and department information. Cover letters were attached to the questionnaire explaining the academic purpose of the survey and ensuring the confidentiality of their responses. In return for their participation, participants who completed the entire survey would receive a reward of around three dollars. We filtered the data of the questionnaire and eliminated invalid questionnaires including wrong answers of quality control questions and regular answers (eight consecutive questions and above with the same answer).

A three-wave survey (with a lag of 1 month in each wave) was used in our study. It has been revealed that a 1-month lag is sufficient to reduce common method bias (Hur et al., 2019; Jiang et al., 2019). The survey was conducted from July to December, 2021. At Time 1, we obtained measures of demographics, responsible leadership, and chronic regulatory focus. A total of 393 questionnaires were distributed and 376 valid questionnaires were returned (response rate 94.91%); At Time 2, we obtained measures of POS-E and green self-efficacy. 358 valid questionnaires were returned (response rate 95.21%); At Time 3, we obtained measure of EPB, and 319 valid questionnaires were returned finally (response rate 89.11%). The overall response rate was 81.17%. The non-response bias analysis showed that there was no significant difference between the dropped sample and the retained sample at time 3 in terms of gender (*t* = 0.30, *p* = 0.78), age (*t* = −0.23, *p* = 0.37), education (*t* = 0.18, *p* = 0.36), tenure (*t* = −0.46, *p* = 0.67) and relationship duration (*t* = 0.39, *p* = 0.52). We also compared early respondents (i.e., participants in T1) with late respondents (i.e., participants in T2) in terms of demographic characteristics (Armstrong and Overton, 1977). *T*-tests showed non-significant differences between the three waves of respondents in terms of demographic characteristics. Therefore, the results of the test confirm that non-response bias is not an issue.

Among the 319 valid questionnaires, female employees accounted for 42.9% and male employees 57.1%. Their average age was 35.64 years (SD = 6.21), average tenure was 5.01 years (SD = 3.62) and average relationship duration was 4.68 years (SD = 3.89). Employees with undergraduate degree or above accounted for 87.5%, and employees without undergraduate degree accounted for 12.5%. The respondents have a wide range of characteristics and coverage, which are suitable for further empirical analysis.

### 3.2 Measures

The measurement items of all constructs in our study were derived from the confirmed mature scale. In order to ensure the reliability and validity of the measurement items in the Chinese context, we followed the standard translation and back-translation procedures. We invited six experts to evaluate the content validity, including experienced scholars and enterprise employees. The results showed that the content validity index was greater than 80%, ensuring the requirements of content validity. To reduce the influence of social desirability, all constructs in this study (except for demographic variables) were measured using a six-point Likert-type scale (1 = strongly disagree to 6 = strongly agree). Appendix Table A1 shows the detailed measurement items.

Responsible leadership was assessed with the 5-item scale developed by Voegtlin (2011). A sample item included “My direct supervisor weighs different stakeholder claims before making a decision”. The Cronbach’s alpha coefficient was 0.799.

Green self-efficacy was measured with the 6-item scale developed by Chen et al. (2015). A sample item was “I think I can perform effectively on environmental missions.” The Cronbach’s alpha coefficient was 0.807.

Perceived organizational support toward the environment (POS-E) was measured with the 5-item scale developed by Lamm et al. (2015). A sample item was “My organization does not care about whether I behave in a sustainable manner or not”. The Cronbach’s alpha coefficient was 0.848.

Pro-environmental behaviors was measured with the 6-item scale developed by Bissing-Olson et al. (2013). A sample item was “I adequately completed assigned duties in environmentally-friendly ways”. The Cronbach’s alpha coefficient was 0.845.

Chronic regulatory focus was measured with the 18-item scale developed by Lockwood et al. (2002), composed of a 9-item scale of promotion focus and a 9-item scale of prevention focus. A sample item for promotion focus was “I frequently imagine how I will achieve my hopes and aspirations”, the Cronbach’s alpha coefficient was 0.893. A sample item for promotion focus was “I am focused on preventing negative events in my life”, the Cronbach’s alpha coefficient was 0.889.

To minimize the estimation bias caused by missing variables, we controlled the demographic variables of gender, age, education level, organizational tenure and relationship duration with leaders of the respondents in this study based on the previous literatures (Robertson and Barling, 2013; Kim et al., 2017; Paillé et al., 2019a).

## 4. Analysis and results

### 4.1 Common method bias

Considering that all the variables in our study were self-reported by employees, it was necessary to assess the risk of common method bias. We used the Harman single-factor test method, and the results showed that a total of seven factors had eigenvalues greater than 1, and the first factor explained 20.71% of the total variance, not exceeding the recommended value of 40%. Therefore, the common method bias problem in our study was not serious.

### 4.2 Confirmatory factor analysis

Confirmatory factor analysis was conducted using MPLUS 8.3 software to test the reliability and validity of the model. As shown in Table 1, the six-factor model had satisfactory fit indices (χ^2^/df = 1.564, CFI = 0.928, TLI = 0.922, RMSEA = 0.042, and SRMR = 0.050) and outperformed other alternative models. Therefore, there was good discriminant validity among the six variables involved in this study.

**TABLE 1:** Confirmatory factor analysis results for model comparisons.

Model types	χ^2^	*df*	χ^2^/*df*	RMSEA	CFI	TLI	SRMR
Six-factor model (RL, GSE, POS-E, EPB, PVF, and PMF)	1,134.260	725	1.564	0.042	0.928	0.922	0.050
Five-factor model (RL, GSE, POS-E, EPB, and PVF + PMF)	1,451.955	730	1.989	0.056	0.872	0.864	0.057
Four-factor model (RL, GSE + POS-E, EPB, and PVF + PMF)	1,904.443	734	2.595	0.071	0.793	0.780	0.073
Three-factor model (RL + POS-E, GSE + EPB, and PVF + PMF)	2,127.426	737	2.887	0.077	0.754	0.740	0.080
Two-factor model (RL + PVF + PMF and EPB + GSE + POS-E)	2,562.901	739	3.468	0.088	0.677	0.659	0.114
One-factor model (RL + GSE + POS-E + EPB + PVF + PMF)	4,619.778	740	6.243	0.128	0.314	0.277	0.194

RL, responsible leadership; GSE, green self-efficacy; POS-E, perceived organizational support toward the environment; EPB, employees pro-environmental behavior; PMF, promotion focus; PVF, prevention focus.

Table 2 shows the factor loadings, average variance extracted (AVE) and the composite reliability (CR). According to Fornell and Larcker (1981), the AVE values met the recommended threshold of 0.50, indicating that the scales have acceptable convergent validity. The CR values met the recommended threshold of 0.8, ensuring internal consistency of measures. The square root of AVE was greater than the correlation coefficient, further proving that the variables have good discriminant validity.

**TABLE 2:** Measurement validity assessment.

Constructs	No. of items	Loadings range	AVE	CR
Responsible leadership	5	[0.671–0.754]	0.527	0.848
POS-E	5	[0.690–0.761]	0.530	0.849
Green self-efficacy	6	[0.696–0.793]	0.532	0.872
EPB	6	[0.611–0.813]	0.513	0.861
Promotion focus	9	[0.482–0.764]	0.500	0.896
Prevention focus	9	[0.637–0.783]	0.516	0.905

### 4.3 Descriptive statistics

First, we used Mardia’s measures (Mardia, 1970) for multivariate skewness. Mardia’s normalised estimate of multivariate skewness was 4.69 (*p* > 0.05). According to Byrne (2006) and Cain et al. (2017), the result showed that the joint distribution of variables hasn’t significant skewness.

Then, we used SPSS software to calculate the means, standard deviations, and correlation coefficients between variables. As Table 3 shows, responsible leadership was positively related to green self-efficacy (β = 0.438, *p* < 0.01), POS-E (β = 0.229, *p* < 0.01), EPB (β = 0.553, *p* < 0.01). Green self-efficacy (β = 0.534, *p* < 0.01), POS-E (β = 0.497, *p* < 0.01) and EPB were significantly and positively correlated. Green self-efficacy was positively related to POS-E (β = 0.263, *p* < 0.01). The results provided preliminary support for the theoretical hypotheses of our study.

**TABLE 3:** Descriptive statistics and correlations of variables.

	M	SD	1	2	3	4	5	6	7	8	9	10
(1) Gender	0.57	0.496										
(2) Age	35.64	6.212	–0.099									
(3) Education	2.95	0.562	0.074	–0.085								
(4) Tenure	5.01	3.618	−0.168**	0.674**	–0.050							
(5) Relationship duration	4.68	3.887	–0.107	0.518**	–0.016	0.693**						
(6) RL (Time 1)	4.80	0.678	–0.042	0.012	0.055	0.046	0.069					
(7) PMF (Time 1)	4.63	0.797	–0.028	–0.028	–0.070	–0.044	–0.034	–0.049				
(8) PVF (Time 1)	3.17	0.927	–0.018	–0.027	0.149**	–0.001	–0.034	0.103	−0.657**			
(9) GSE (Time 2)	4.90	0.639	–0.077	0.096	–0.034	0.117*	0.086	0.438**	0.013	–0.013		
(10) POS-E (Time 2)	4.03	0.929	−0.116*	–0.085	0.034	–0.011	0.008	0.229**	–0.005	0.010	0.263**	
(11) EPB (Time 3)	4.62	0.725	−0.141*	0.055	0.052	0.123*	0.102	0.553**	0.075	0.001	0.534**	0.497**

*N* = 319; *p < 0.05, **p < 0.01, ***p < 0.001 (two tailed).

### 4.4 Hypothesis testing

We conducted a hierarchical regression analysis with SPSS software, and Bootstrap test for indirect effects using the PROCESS macro.

(1) Analysis of the effect of responsible leadership on EPB. As shown in Table 4, Model 4 added independent variables based on Model 3, the results showed that responsible leadership was positively related to EPB (β = 0.580, *p* < 0.001). Thus, H1 was supported.

**TABLE 4:** Hierarchical regression models: The mediating effect of green self-efficacy (GSE) and perceived organizational support toward the environment (POS-E).

Variable types	GSE	POS-E	EPB			
	Model 1	Model 2	Model 3	Model 4	Model 5	Model 6
Intercept	2.983*** (0.326)	2.965*** (0.511)	4.407*** (0.303)	1.775*** (0.34)	0.578*** (0.354)	0.879** (0.319)
**Control variables**						
Gender	−0.053 (0.066)	−0.213* (0.104)	−0.187* (0.082)	−0.156* (0.069)	−0.135* (0.064)	−0.092 (0.062)
Age	0.036 (0.054)	−0.152 (0.085)	−0.040 (0.068)	−0.022 (0.057)	−0.037 (0.052)	0.024 (0.051)
Education	−0.055 (0.058)	0.034 (0.091)	0.083 (0.072)	0.043 (0.06)	0.065 (0.056)	0.032 (0.054)
Tenure	0.062 (0.068)	0.037 (0.107)	0.104 (0.085)	0.096 (0.071)	0.071 (0.066)	0.085 (0.064)
Relationship duration	−0.028 (0.079)	0.046 (0.125)	0.046 (0.099)	−0.006 (0.083)	0.005 (0.077)	−0.020 (0.074)
**Independent variable**						
RL	0.412*** (0.048)	0.303*** (0.075)		0.580*** (0.05)	0.415*** (0.051)	0.489*** (0.046)
**Mediating variables**						
GSE					0.401*** (0.055)	
POS-E						0.302*** (0.034)
*R* ^2^	0.207	0.076	0.036	0.328	0.427	0.467
ΔR^2^				0.292	0.099	0.139

Numbers in parentheses are standard errors; *N* = 319; *p < 0.05, **p < 0.01, ***p < 0.001. Variance inflation factor (VIF) is between 1.020 and 2.654, so there is no serious multicollinearity problem between variables.

(2) Analysis of the serial multiple mediating effects of POS-E and green self-efficacy. Model 1 and Model 2 showed that responsible leadership had a significantly positive effect on green self-efficacy (β = 0.412, *p* < 0.001) and POS-E (β = 0.303, *p* < 0.001), respectively. According to Model 5, when responsible leadership and green self-efficacy were simultaneously placed in the regression equation for EPB, the role of responsible leadership (β = 0.415, *p* < 0.001) and green self-efficacy (β = 0.401, *p* < 0.001) were significant. Therefore, green self-efficacy played a partial mediating role. According to Model 6, when responsible leadership and POS-E were simultaneously placed in the regression equation for EPB, the role of responsible leadership (β = 0.489, *p* < 0.001) and POS-E (β = 0.302, *p* < 0.001) were significant. Therefore, POS-E played a partial mediating role. To more accurately test the mediating role of POS-E and green self-efficacy, we used the bootstrapping method to test the indirect effect hypotheses. As shown in Table 4, the indirect effect through green self-efficacy was significant (β = 0.120, 95% CI = [0.066, 0.183]), in support of H2. The indirect effect through POS-E was significant (β = 0.080, 95% CI = [0.040, 0.124]), in support of H3. The serial mediating effect through POS-E and green self-efficacy was significant (β = 0.012, 95% CI = [0.003, 0.026]), H4 was supported.

We further compared the above mediating effects. As shown in Table 5, the difference between the two independent mediating effects was not significant (95% CI = [−0.122, 0.039]), indicating that the independent mediation effects of POS-E and green self-efficacy were comparable. However, both independent mediating effects of POS-E and green self-efficacy were greater than the serial mediating effect (β = 0.068, 95% CI = [0.032, 0.110]; β = 0.109, 95% CI = [0.055, 0.171]).

**TABLE 5:** Bootstrapping tests for mediating effect.

Model pathways	Effect	SE	95% CI	
			LL	UL
Direct effect	0.368	0.047	0.148	0.281
Total indirect effect	0.212	0.034	0.148	0.281
M1:RL → POS-E → EPB	0.080	0.022	0.040	0.124
M2:RL → GSE → EPB	0.120	0.030	0.066	0.183
M3:RL → POS-E → GSE → EPB	0.012	0.006	0.003	0.026
D1 = M1 − M2	−0.041	0.041	−0.122	0.039
D2 = M1 − M3	0.068	0.020	0.032	0.110
D3 = M2 − M3	0.109	0.030	0.055	0.171

CI, confidence interval; LL, lower limit; UL, upper limit.

**FIGURE 2:**
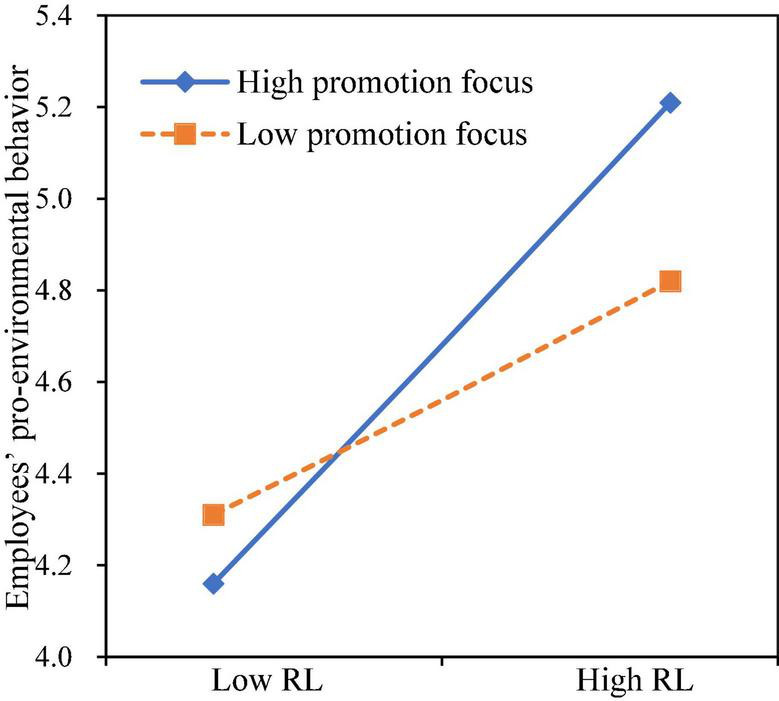
Moderating effect of promotion focus.

(3) Analysis of the moderating effect of regulatory focus. As shown in Table 6, Model 8 added the interaction of responsible leadership with promotion focus based on Model 7, the results presented that responsible leadership interacted with promotion focus to affect EPB (β = 0.251, *p* < 0.001). The moderating effect was illustrated in Figure 2. Simple slope analysis indicated that the impact of responsible leadership on EPB was stronger (β = 0.762, *p* < 0.001) when promotion focus was at a high level (mean + 1 SD), the impact was relatively weaker (β = 0.362, *p* < 0.001) when promotion focus was at a low level (mean−1 SD), in support of H5.

**TABLE 6:** Hierarchical regression models: The moderating effect of promotion focus (PMF) and prevention focus (PVF).

Variable types	EPB			
	Model 7	Model 8	Model 9	Model 10
Intercept	1.260** (0.407)	4.544*** (0.248)	1.877 (0.347)	4.550*** (0.251)
**Control variables**				
Gender	−0.151 (0.069)	−0.142* (0.067)	−0.159* (0.069)	−0.162* (0.068)
Age	−0.022 (0.056)	−0.017 (0.055)	−0.024 (0.057)	−0.015 (0.056)
Education	0.052 (0.060)	0.036 (0.059)	0.055 (0.061)	0.042 (0.060)
Tenure	0.101 (0.071)	0.113 (0.069)	0.100 (0.071)	0.111 (0.070)
Relationship duration	−0.006 (0.082)	−0.022 (0.081)	−0.012 (0.083)	−0.016 (0.082)
**Independent variable**				
RL	0.585*** (0.050)	0.562*** (0.048)	0.587*** (0.050)	0.546*** (0.051)
**Moderating variables**				
PMF	0.096* (0.042)	0.078 (0.041)		
PVF			−0.051 (0.037)	−0.045 (0.036)
**Interactions**				
PMF × RL		0.251*** (0.066)		
PVF × RL				−0.185*** (0.057)
R^2^	0.339	0.368	0.332	0.354
ΔR^2^	0.011	0.029	0.004	0.022

Numbers in parentheses are standard errors; *N* = 319; *p < 0.05, **p < 0.01, ***p < 0.001. Variance inflation factor (VIF) is between 1.021 and 2.651, so there is no serious multicollinearity problem between variables.

**FIGURE 3:**
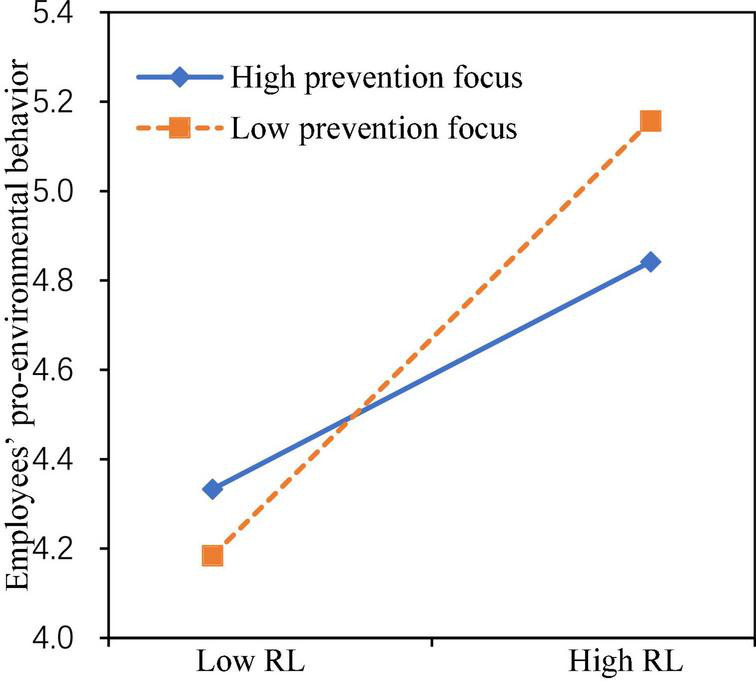
Moderating effect of prevention focus.

Model 10 added the interaction of responsible leadership with prevention focus based on Model 9, the results showed that responsible leadership interacted with prevention focus to predict EPB (β = −0.185, *p* < 0.001). The moderating effect was illustrated in Figure 3. Simple slope analysis showed that the impact of responsible leadership on EPB was stronger (β = 0.717, *p* < 0.001) when prevention focus was at a low level (mean−1 SD), the impact was relatively weaker (β = 0.375, *p* < 0.001) when prevention focus was at a high level (mean + 1 SD). Thus, H6 received support.

**FIGURE 4:**
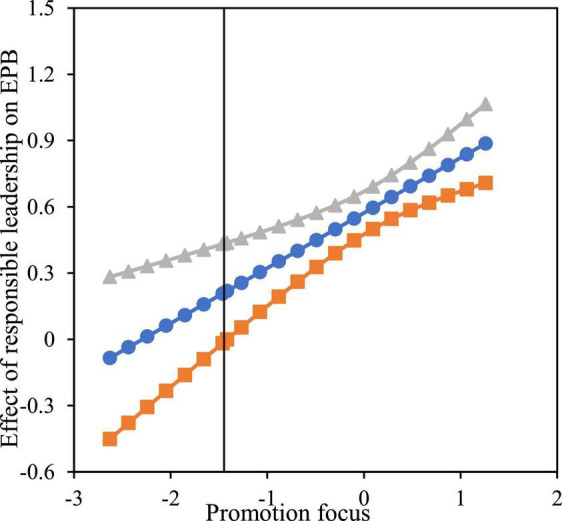
Moderating effect of promotion focus (J-N chart).

**FIGURE 5:**
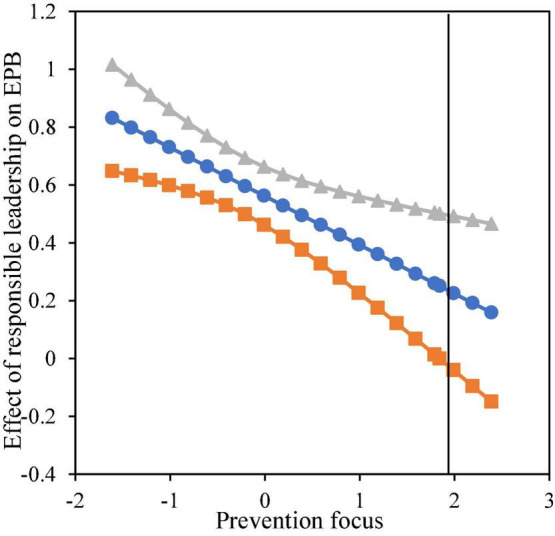
Moderating effect of prevention focus (J-N chart).

Furthermore, we used the Johnson-Neyman technique to identify the regions in the range of the moderator variable where the effect of responsible leadership on EPB was statistically significant and not significant. Figures 4, 5 showed the moderating effect range of the promotion focus and prevention focus respectively. In the 95% confidence interval, when the promotion focus (centralization) was higher than - 1.416, the positive moderating effect was significant; When the prevention focus (centralization) was lower than 1.989, the negative moderating effect was significant.

## 5. Discussion and implications

Integrating the resource accumulation perspective and the JD-R model, we developed a conceptual model to explain how and when responsible leadership evokes EPB. We analyzed the serial multiple mediating roles of POS-E and green self-efficacy between responsible leadership and EPB in a Chinese organizational context, and explored the moderating roles of promotion focus and prevention focus. The conclusions are as follows: First, responsible leadership has a significant positive effect on EPB. This is consistent with previous studies (Afsar et al., 2020; Zhang J. W. et al., 2021). Second, responsible leadership also further positively influences EPB by increasing POS-E and green self-efficacy of employees, and POS-E and green self-efficacy play a serial mediating role between responsible leadership and EPB. Previous studies have also confirmed the impact of POS-E and green self-efficacy on EPB (Lamm et al., 2015; Faraz et al., 2021; Paillé and Valeau, 2021). Differently, we further confirmed that responsible leadership can affect POS-E and green self-efficacy of employees, thereby promoting them to adopt pro-environmental behaviors. Third, employees’ promotion focus positively moderates the effect of responsible leadership on EPB, while employees’ prevention focus negatively moderates the effect of responsible leadership on EPB. This result also supports the view that responsible leadership may cause resource loss to employees (Zhu et al., 2021), and employees with different characteristics will have different perceptions of responsible leadership.

### 5.1 Theoretical implications

First, our research extends the existing literatures on the antecedents of EPB. EPB needs the guarantee of external environmental (job) resources and the support of personal resources, and only when external environmental (job) resources and personal resources work simultaneously can EPB be better motivated (Raineri and Paillé, 2016). However, most of the previous studies have explored the influence of a certain type of resources on EPB, ignoring the joint influence of environmental (job) resources and personal resources. Our research integrates leadership, job resources, individual psychological resources, and individual characteristic differences, and explores the influence of these factors on EPB.

Second, we combine the resource accumulation perspective and the JD-R model to provide a new research perspective on how responsible leadership promotes pro-environmental behaviors and also extend the research on the influence mechanisms of responsible leadership. Previous studies have concentrated on self-determination theory (e.g., Graves et al., 2013), norm-activation model (e.g., Afsar et al., 2016), social learning theory (e.g., Faraz et al., 2021), and social exchange theory (e.g., Afsar et al., 2016) to explain why EPB arise, ignoring the importance of resources in the process. In addition, previous studies have explored the positive effects brought by responsible leadership in terms of emotion (Doh et al., 2011), organizational commitment (Haque et al., 2019b), and organizational climate (Yasin, 2021), ignoring the resource perspective. Based on the JD-R model, our study discovered the serial multiple mediating roles of two types of resources, POS-E and green self-efficacy, between responsible leadership and EPB, as well as the moderating role of regulatory focus as individual difference, which provides a novel perspective on how responsible leadership affects EPB and enriches the existing research.

Third, our study extends the understanding to the contingency relationship between responsible leadership and EPB by demonstrating the moderating role of chronic regulatory focus. Although several scholars have recognized that responsible leadership may cause work stress and resource depletion for employees (Zhu et al., 2021), the studies on boundaries of responsible leadership effectiveness were still scant. Our study found that promotion focus reinforced the positive effect of responsible leadership on EPB, while prevention focus weakened the effect. Therefore, this result provides new insights into understanding how to improve the effectiveness of responsible leadership in Chinese organizational contexts, reveals the reinforcement mechanism of EPB in terms of individual differences.

Finally, we apply JD-R theory to the research field of EPB, which broadens the application scope of this theory and provides evidence for its explanatory power in the new research field. The job resources (POS-E), individual psychological resources (green self-efficacy) and individual characteristics differences (regulatory focus) are used to explain the relationship between responsible leadership and EPB, which not only supports the view of JD-R model, but also widens the mediation mechanism and moderation mechanism of JD-R model in the research of responsible leadership and EPB.

### 5.2 Practical implications

First, considering the impact of responsible leadership on EPB, companies should train their current leaders and cultivate responsible leaders. For example, companies can help leaders clarify their roles and behaviors by formulating specific rules of duty (Maak and Pless, 2006) and conducting courses related to management responsibility awareness (Voegtlin et al., 2012), so as to cultivate and develop responsible leaders. Voegtlin et al. (2019) found that empathy, positive affect and self-transcendence values orientation can promote responsible leadership. Therefore, companies can conduct relevant courses for managers to stimulate these individual characteristics, and then cultivate responsible leaders. In addition, when selecting managers, companies can evaluate their values, abilities, sense of responsibility, and environmental awareness, and choose those who are more likely to become responsible leaders.

Second, Considering that POS-E and green self-efficacy can affect EPB, managers should pay attention to the impact of corporate decisions on the environment, show more environmental protection behaviors in their daily management work, and regularly organize employees to participate in environmental protection activities, so that employees can realize the importance and support of the companies to environmental protection; Managers should pay more attention to employees’ inner state, understand employees’ needs and difficulties in environmental protection practice in time, provide training and assistance for employees, and stimulate employees’ green self-efficacy.

Third, considering that regulatory focus of employees can affect the effectiveness of responsible leadership, companies can conduct personality assessment and questionnaire surveys to fully understand the chronic regulatory focus of employees, and assign tasks and carry out work according to their personality, so that responsible leadership effectiveness can be enhanced. For positions with high expectations of EPB, companies should employ promotion-focused employees through scientific and standardized recruitment procedures to better stimulate EPB; Companies also can cultivate promotion-focused employees through training.

### 5.3 Limitations and future research directions

First, although our data were gathered through a three-wave survey, they were all obtained by employees’ self-report, and this may lead to common method bias. Therefore, it can be considered in future research that leaders and employees fill out the questionnaires separately to decrease common method bias.

Second, although our study examined POS-E and green self-efficacy as mediating mechanisms, future research could explore whether other resources can serve as mediating mechanisms between responsible leadership and EPB. For example, social capital (Harris et al., 2018), personal learning (Jiang et al., 2015), and psychological ownership (Sun et al., 2019).

Third, our study only considered the moderating effect of chronic regulatory focus. Individuals’ cognition of leadership may also be affected by other characteristics of employees and environmental factors. Therefore, other individual characteristic factors (e.g., proactive personality) or environmental factors (e.g., organizational environmental atmosphere) can also be considered as moderating variables in future research.

Finally, our study deeply discussed the motivation effect of job resources and personal resources on EPB from the resource path of JD-R model, but didn’t consider the impact of job demands and personal demands. Future research can further discuss the mediating or moderating role of job demands and personal demands between responsible leadership and EPB.

## 6. Conclusion

Based on the resource accumulation perspective and the JD-R model, we have theoretically established and empirically tested a conceptual model linking responsible leadership, POS-E, green self-efficacy, chronic regulatory focus and EPB. This study not only extends the research on the antecedents of EPB and the impact mechanism of responsible leadership, but also enriches the theoretical connotation and application scope of the JD-R model. Our results also have important implications for companies and managers that must enhance employees’ POS-E and green self-efficacy through the practice of responsible leadership and induce employees’ promotion focus.

## Data availability statement

The raw data supporting the conclusions of this article will be made available by the authors, without undue reservation.

## Author contributions

YZ: conceptualization, methodology, formal analysis, and writing—original draft preparation. YZ and YN: validation. HH: data curation. YN, HH, and DL: writing—review and editing. YZ, YN, HH, and DL: investigation, reading, and agreeing to the published version of the manuscript. All authors contributed to the article and approved the submitted version.

## Appendix

**TABLE A1:** Scale items.

Constructs	Items
Responsible leadership	My leader demonstrates awareness of the relevant stakeholder claims
	My leader considers the consequences of decisions for the affected stakeholders
	My leader involves the affected stakeholders in the decision-making process
	My leader weighs different stakeholder claims before making a decision
	My leader tries to achieve a consensus among the affected stakeholders
POS-E	I feel that I am able to behave as sustainably as I want to at the organization where I currently work
	My organization does not care about whether I behave in a sustainable manner or not
	My organization provides an incentive for me to reduce the use of non-renewable resources
	I do not feel that I make a positive environmental impact through work at my organization
	My actions toward sustainability are appreciated by my organization
Green self-efficacy	I feel I can succeed in accomplishing environmental ideas
	I can achieve most of environmental goals
	I feel competent to deal effectively with environmental tasks
	I can perform effectively on environmental missions
	I can overcome environmental problems
	I can find out creative solutions to environmental problems
EPB	I adequately completed assigned duties in environmentally-friendly ways
	I fulfilled responsibilities specified in my job description in environmentally-friendly ways
	I performed tasks that are expected of me in environmentally-friendly ways
	I took a chance to get actively involved in environmental protection at work
	I took initiative to act in environmentally-friendly ways at work
	I did more for the environment at work than I was expected to
Promotion focus	I frequently imagine how I will achieve my hopes and aspirations
	I often think about the person I would ideally like to be in the future
	I typically focus on the success I hope to achieve in the future
	I often think about how I will achieve academic success
	My major goal in school right now is to achieve my academic ambitions
	I see myself as someone who is primarily striving to reach my “ideal self”—to fulfill my hopes, wishes, and aspirations
	In general, I am focused on achieving positive outcomes in my life
	I often imagine myself experiencing good things that I hope will happen to me
	Overall, I am more oriented toward achieving success than preventing failure
Prevention focus	In general, I am focused on preventing negative events in my life
	I am anxious that I will fall short of my responsibilities and obligations
	I often think about the person I am afraid I might become in the future
	I often worry that I will fail to accomplish my career goals
	I often imagine myself experiencing bad things that I fear might happen to me
	I frequently think about how I can prevent failures in my life
	I am more oriented toward preventing losses than I am toward achieving gains
	My major goal right now is to avoid becoming a career failure
	I see myself as someone who is primarily striving to become the self I “ought” to be—to fulfill my duties, responsibilities, and obligations
